# Mutation Spectrum of *ADAMTS13* Gene in Patients with Upshaw–Schulman Syndrome (USS) in Russia

**DOI:** 10.3390/ijms27104643

**Published:** 2026-05-21

**Authors:** Julia Poznyakova, Olesya Pshenichnikova, Elizaveta Klebanova, Gennadiy Galstyan, Vadim Surin

**Affiliations:** 1 Laboratory of Genetic Engineering, National Medical Research Center for Hematology, Novy Zykovski Lane 4a, 125167 Moscow, Russia; y.poznyakova@mail.ru (J.P.); vadsurin@mail.ru (V.S.); 2 ICU and Intensive Care Department, National Medical Research Center for Hematology, Novy Zykovski Lane 4a, 125167 Moscow, Russia; klebanova.e@blood.ru (E.K.); galstyan.g@blood.ru (G.G.)

**Keywords:** thrombotic thrombocytopenic purpura, Upshaw-Schulman syndrome, *ADAMTS13* gene, mutation spectrum

## Abstract

Upshaw–Schulman syndrome (USS) is a rare inherited autosomal recessive thrombotic microangiopathy affecting less than 1/1,000,000 individuals. It is a congenital form of thrombotic thrombocytopenic purpura (TTP) caused by ADAMTS13 protease deficiency because of mutations in the *ADAMTS13* gene. USS is characterized by the formation of platelet thrombi in the microcirculation, accompanied by hemolytic anemia, thrombocytopenia, and clinical and laboratory signs of renal and neurological failure. The aim of this study was to describe the *ADAMTS13* gene mutation spectrum in the Russian population. We analyzed the *ADAMTS13* gene in 45 unrelated patients with TTP of unknown origin. DNA was extracted from blood cells using the phenol-chlorophorm method, and all exons of the gene were investigated using Sanger sequencing. In 15 out of 45 patients, we identified 20 different variants associated with USS, including two frameshift, two variants affecting the splice site, one nonsense and fifteen missense mutations. Eight out of those mutations were previously undescribed. Tree variants were revealed more than once: p.Arg1060Trp (7 patients), p.Glu1326ArgfsTer6 (7 patients) and p.Cys1067SerfsTer30 (3 patients). Variants (p.Arg1060Trp) and p.Glu1326ArgfsTer6 prevailed in the global population; however, p.Cys1067SerfsTer30 was not previously described in the European population. Our results expand the existing knowledge of the molecular basis of USS and may contribute to improved genetic diagnostics in Russia.

## 1. Introduction

Upshaw–Schulman syndrome (USS) (MIM:274150), also known as congenital thrombotic thrombocytopenic purpura (cTTP), is a rare autosomal recessive disorder caused by mutations in the *ADAMTS13* gene. *ADAMTS13* located on chromosome 9 (9q34), spans approximately 45,066 base pairs, and contains 29 exons [1]. It encodes metalloproteinase ADAMTS13 (A Disintegrin And Metalloproteinase with ThromboSpondin type 1 motif, member 13), which was first described in 2001.

ADAMTS13 consists of 1427 amino acid residues and is organized into several functional domains, including an N-terminal metalloproteinase domain, disintegrin, thrombospondin type 1 (TSP1) repeat, cysteine-rich and spacer domains, as well as seven additional TSP-1 repeats and two C-terminal CUB domains. The two C-terminal CUB domains are unique within the ADAMTS family [2].

ADAMTS13 is a specific protease responsible for the cleavage of ultra-large multimers of von Willebrand factor (UL-VWF) at the Tyr1605-Met1606 bond [3,4]. VWF is a large multimeric glycoprotein essential for platelet-plug formation. It acts as an adhesion protein, which redirects circulating platelets to the vascular injury sites. Deficiency of ADAMTS13 activity leads to the accumulation of uncleaved UL-VWF. That, in turn, results in increased platelet aggregation and adhesion in microcirculation, causing the formation of platelet-rich thrombi [5]. Cleavage of UL-VWF is a complex and extremely specific process that requires numerous interactions between distinct domains of both VWF and ADAMTS13 [2].

USS is characterized by consumptive thrombocytopenia, microangiopathic hemolytic anemia (MAHA) and dysfunction of various organs and systems, typically including neurologic, cardiac, gastrointestinal and renal involvement [5]. The disease is diagnosed more frequently in children, accounting for approximately 30% of all TTP cases in infants, compared with fewer than 5% of all TTP cases in adults. Clinical manifestation at the debut of the disease differs with the age of onset. Childhood-onset USS typically begins in the neonatal period and is characterized by hematological manifestations and jaundice. In contrast, adult-onset USS is often first identified during pregnancy [6,7]. During the second and third trimesters of pregnancy, healthy women demonstrate a marked increase in VWF release and a decrease in ADAMTS13 activity [8]. Under these conditions, fully functional ADAMTS13 is essential, and any impairment in its activity may lead to the development of clinical manifestations. This may explain the predominance of adult-onset USS cases associated with pregnancy.

The diagnosis of TTP is verified by detecting a severe deficiency (<10%) of plasma ADAMTS13 activity [9]. ADAMTS13 deficiency may be congenital, resulting from mutations in the *ADAMTS13* gene, or acquired, due to the production of anti-ADAMTS13 autoantibodies [10,11]. Immune-mediated TTP represents the more common form of the disease, although it remains rare, with an incidence of approximately 10 cases per 1,000,000 persons per year. In contrast, cTTP is considered an ultra-rare disorder, affecting fewer than 1 per 1,000,000 individuals. Mutations in the *ADAMTS13* gene may impair the secretion of this enzyme and/or reduce its protease activity [12]. The rarity of this disease complicates the understanding of factors behind the diverse and complex clinical presentation. To date, no definitive genotype–phenotype correlation has been identified [2,6,7,13]. Currently, 250 different pathogenic variants (missense, nonsense, splice site mutations and frameshift mutations caused by small deletions and insertions) in the *ADAMTS13* gene have been registered in the HGMD (Human Gene Mutation Database), most of which are missense mutations [14].

The objective of this work was to describe the mutation spectrum of the *ADAMTS13* gene in the Russian population as it had never been done before.

## 2. Results

In 15 out of 45 people (33%), we found gene defects that may affect the expression or activity of ADAMTS13 (Table 1). This group consists of 4 male and 11 female patients.

We identified four patients carrying both ADAMTS13 inhibitors and pathogenic gene variants, as previously described by Camilleri et al. [15] as well as two patients with ADAMTS13 activity slightly above the diagnostic threshold of 10% [9].

A total of 20 different gene variants were identified. Among them were six known pathogenic variants, five functional polymorphisms, one previously described variant of uncertain significance (VUS) c.1261 C>T (p.Arg421Cys) and eight novel variants (Table 2).

### 2.1. Causative Variants

In 15 of the 45 individuals studied, we identified a total of 14 distinct genetic defects, including eight novel variants, that are likely to severely impair ADAMTS13 function and contribute to the development of USS. Thirteen patients were compound heterozygous and two patients were homozygous. One of the identified variants, c.1159G>C (p.Trp387Ser), was previously described in our earlier study [16].

**Figure 1:**
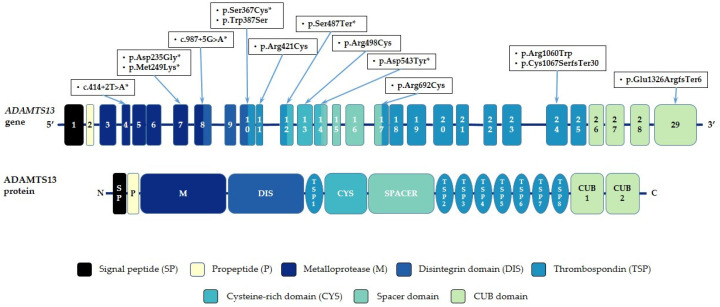
Distribution of different causal variants found among patients with cTTP in Russia. * Previously undescribed variants.

**Figure 2:**
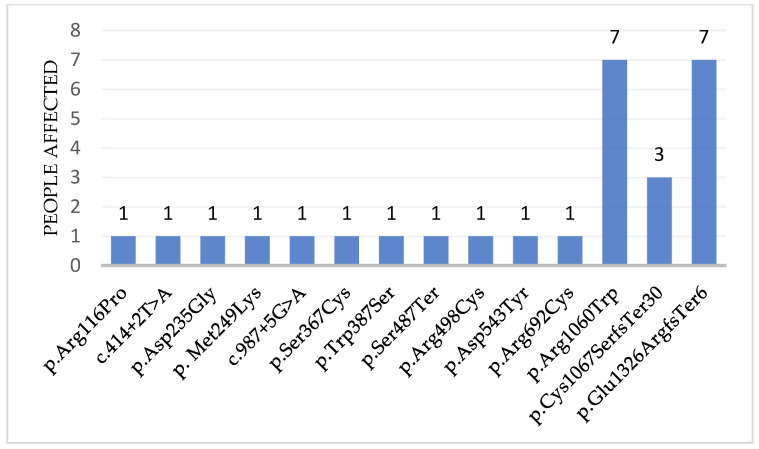
Spectrum of causative variants and their frequencies revealed in Russian patients with USS in this study.

Overall, all found pathogenic variants were distributed uniformly along the gene (Figure 1). The most common type of pathogenic variant in our cohort was missense mutation—we identified 9 of them (64%). In addition, we detected two variants that affect the splicing site, one nonsense mutation, one frame shift deletion and one frame shift insertion. Three pathogenic variants were encountered more than once (Table 1, Figure 2): variants c.3178 C>T (p.Arg1060Trp) and c.3975dup (p.Glu1326ArgfsTer6) were identified in 7 patients (46%) each, variant c.3198_3199del (p.Cys1067SerfsTer30) was revealed in 3 patients (20%).

In 10 out of 11 female patients (91%), the debut of the disease was associated with the first pregnancy. Six of them carried c.3178 C>T (p.Arg1060Trp) variant.

We noted that the c.3178 C>T (p.Arg1060Trp) pathogenic variant was linked with SNP c.3097 G>A (p.Ala1033Thr), and it always occurred in pairs (result of linkage disequilibrium test was D’ = 1.0, *p* < 0.0001).

### 2.2. Previously Undescribed Variants

Eight variants were previously undescribed. Among them there were five missense variant (c.347 G>C (p.Arg116Pro), c.704 A>G (p.Asp235Gly), c.746T>A (p.Met249Lys), c.1100 C>G (p.Ser367Cys) and c.1627 G>T (p.Asp543Tyr)), two variants were disrupting splice sites (c.414+2T>A and c.987+5G>A) and one nonsense variant (c.1461 C>A (p.Ser487Ter)). All of them were found only once each.

The ACMG/AMP Variant Curation Guidelines evaluated missense variants c.347 G>C (p.Arg116Pro) and c.1627 G>T (p.Asp543Tyr) as variants of unknown significance (VUS) (with PM2, PP3, PP4 ACMG criteria).

Three missense variants c.704 A>G (p.Asp235Gly), c.746T>A (p.Met249Lys) and c.1100 C>G (p.Ser367Cys) were assessed as likely pathogenic (with PM2, PP3, PM1, PP4 criteria for all three of them, and additional PM5 criteria for c.704 A>G variant). Variant c.704 A>G (p.Asp235Gly) damages an amino acid residue where other missense changes were determined to be pathogenic (c.703G>T (p.Asp235Tyr) and c.703G>C (p.Asp235His) [2,20,21].

Among the two variants that were disrupting splice sites, one was classified as pathogenic (c.414+2T>A with PM2, PVS1, PP4 criteria) and the other was evaluated as a variant of unknown significance (c.987+5G>A with PM2, PP3, PP4 ACMG criteria).

A variant c.1461 C>A (p.Ser487Ter) is a substitution of C to A leading to the formation of a stop codon, and the ACMG/AMP Variant Curation Guidelines evaluated it as pathogenic (PM2, PVS1, PP4 criteria).

## 3. Discussion

Our study provides an overview of USS in Russia, identifying 20 distinct *ADAMTS13* sequence variants in 12 patients. The spectrum of pathogenic variants in the *ADAMTS13* gene in Russian patients with USS is consistent with that reported in other populations. As in other countries [6,7], these variants were distributed almost uniformly along the gene with a prevalence of missense mutations.

In our cohort, most causative variants were observed once, with the exception of c.3178 C>T (p.Arg1060Trp), c.3975dup (p.Glu1326ArgfsTer6) and c.3198_3199del (p.Cys1067SerfsTer30) (Table 2). Variants c.3178 C>T (p.Arg1060Trp) and c.3975dup (p.Glu1326ArgfsTer6) are well-established recurrent mutations in the global population [7,21]. It was previously shown that c.3975dup (p.Glu1326ArgfsTer6) was specific for patients originating from Central and Northern Europe [22]. In contrast, c.3178C>T (p.Arg1060Trp) has a worldwide distribution and has been reported with relatively high frequency in Europe, Scandinavia, Turkey, and North America [23]. Wide distribution of this mutation can also be explained by its polyphyletic origin because this variant is located in CpG dinucleotide. The third recurrent variant identified in our cohort, c.3198_3199del (p.Cys1067SerfsTer30), has not previously been reported in European populations and has been described in patients from Japan and Saudi Arabia [7,24]. We hypothesize that the presence of this variant in our cohort may reflect historical migration and population admixture, given Russia’s geographical position between these regions.

The high proportion of previously unreported variants in our study (40%) further supports the substantial allelic heterogeneity of *ADAMTS13* [2,6,7]. This observation likely reflects the gene’s extensive coding region and the rarity of the disorder, both of which limit the accumulation of recurrent variants in global databases.

In agreement with previous studies [2,6,7,13], no definitive genotype–phenotype correlation was identified. This phenotypic heterogeneity may be attributable to the influence of functional polymorphisms and additional regulatory mechanisms affecting ADAMTS13 secretion or activity. Several studies have proposed that polymorphisms such as c.1342C>G (p.Gln448Glu), c.19C>T (p.Arg7Trp), and c.1852C>G (p.Pro618Ala) may modulate ADAMTS13 function [6,25,26]. Of these, c.1852C>G (p.Pro618Ala) has been associated with a significant reduction in ADAMTS13 activity, whereas c.1342C>G (p.Gln448Glu) and c.19C>T (p.Arg7Trp) appear to have minimal functional impact. Notably, the combined presence of these variants has been reported to yield ADAMTS13 activity within the normal range [26,27].

A notable mention was Patient AD24, who, along with a well-known pathogenic mutation c.3178 C>T (p.Arg1060Trp), carried a rare variant c.1261 C>T (p.Arg421Cys) and novel missense variant c.347 G>C (p.Arg116Pro). Variant c.347 G>C (p.Arg116Pro) has a low allele frequency (0.00006198% [27]) and assessed as VUS. Variant c.1261 C>T (p.Arg421Cys) has a relatively high allele frequency (0.055% [27]) for a pathogenic variant in the *ADAMTS13* gene, as they tend to be single time events with exceptions of several pathogenic mutations [7]. However, Pagliari et al. [28] in their work showed that this variant has a drastic effect on ADAMTS13 expression. Using the ACMG/AMP Variant Curation Guidelines, we evaluated c.1261 C>T (p.Arg421Cys) variant as likely pathogenic (with PM2, PS3, PP4 criteria). Considering all mentioned above, we conclude that c.1261 C>T (p.Arg421Cys) variant has a more negative effect on ADAMTS13 activity than c.347 G>C (p.Arg116Pro), even though most in silico predictors say the opposite.

Since our Center specializes in the treatment of adult patients, our cohort was biased toward individuals with adult-onset USS (78%), limiting our ability to draw conclusions regarding age-specific groups. Nevertheless, several findings in our study are consistent with previous reports. Joly et al. [6] demonstrated that homozygous mutations are more frequent in childhood-onset USS than in adult-onset disease. In agreement with these observations, the proportion of homozygous patients in our cohort was relatively low (2 of 15 patients, 13%). On the other hand, in general, child-onset of USS can be provoked by variants that severely affect ADAMTS13 activity and/or secretion in either homozygous or compound heterozygous state. In their studies, Camilieri et al. [15] and Joly et al. [6] described an association of pathogenic variant c.3178 C>T (p.Arg1060Trp) associated with an adult onset of USS. Consistent with these findings, this variant was also among the most frequent mutations identified in our predominantly adult-onset cohort.

The frequency of genetic variants in the general population does not necessarily reflect their prevalence among affected individuals. In our cohort of patients with cTTP, we observed that some variants with very low population frequencies were recurrent, whereas others with higher frequencies appeared only once. For instance, the c.3975dup variant, which is one of the most common pathogenic variants associated with USS, has a gnomAD frequency of 0.005, and the c.3198_3199del variant identified in our cohort has a frequency of 0.001057%. In contrast, the c.1261C>T (p.Arg421Cys) variant, which has a relatively higher population frequency of 0.055% in gnomAD, was detected only once in our study population. The relatively high prevalence of rare variants and the scarcity of more common variants in our cohort are consistent with previous reports suggesting that rare SNVs may demonstrate population-specific distribution patterns [28,29].

The study was designed to enable the identification of rare USS presentations previously described in the literature, including patients carrying both ADAMTS13 inhibitors and pathogenic *ADAMTS13* variants, as reported by Camilleri et al. [15], as well as cases of cTTP with unusually high ADAMTS13 activity described by Subhan et al. [30]. Among patients harboring pathogenic variants in the *ADAMTS13* gene, we identified four individuals with detectable ADAMTS13 inhibitors (three patients: 0.6 BU; one patient: 0.7 BU; reference value < 0.4 BU) and two patients with ADAMTS13 activity of 12%. Although these findings do not fully meet the diagnostic criteria proposed by Scully et al. [9], the observed values were only marginally above the established thresholds. Therefore, we consider these deviations more likely to reflect methodological variability rather than true biological differences.

In our country, awareness of the cTTP, also known as USS, in adults began to spread only recently. We still do not have a unified patient register, which leads to severe difficulties in obtaining consistent clinical data and any information about relatives. As a result, this study mostly focused on laboratory findings. We further plan to conduct functional assays on the eight novel variants identified in this study to confirm their pathogenicity. Continued research is essential to expand the cohort of patients with USS and enable more in-depth investigation of genotype-phenotype correlations.

We report herein the results of molecular genetic analysis in patients with USS in Russia. Overall mutation spectrum of the *ADAMTS13* gene reflects tendencies revealed in other populations. Major mutations in our population were c.3178 C>T (p.Arg1060Trp), c.3198_3199del (p.Cys1067SerfsTer30) and c.3975dup (p.Glu1326ArgfsTer6). This is the first report of *ADAMTS13* genetic analysis in Russia, and it contributes to local and global knowledge of molecular mechanisms of USS that could improve diagnostics of this disorder in our country.

## 4. Materials and Methods

Due to the difficulties of USS diagnosis and lack of ADAMTS13 activity tests and Anti-ADAMTS13 IgG assays in the distant regions of Russia, for this work, we evaluated patients with suspected TTP. In our country, ADAMTS13 activity test and ADAMTS-13 inhibitor assays are available only in our Center, thus provides us with a unique opportunity to collect enough data from patients with such a rare congenital disease.

All patients involved in the study were directed to the National Medical Research Center of Hematology (Moscow, Russia) for verification of TTP, and then referred to the laboratory for genetic screening. Unfortunately, not all patients were able to afford a cross-country trip, and only a blood sample was available for testing. All patients or their guardians gave their written informed consent. The present work was carried out in accordance with the Code of Ethics of the World Medical Association (Declaration of Helsinki) for experiments involving humans.

TTP was defined by MAHA, thrombocytopenia, normal coagulation screen, raised lactate dehydrogenase, schistocytes on blood film, clinical or objective evidence of organ ischemia. In plasma samples, ADAMTS13 activity was measured with enzyme-linked immunosorbent assay (ELISA) using Technozym ADAMTS-13 kits (Technoclone GmbH, Vienna, Austria) in accordance with the manufacturer’s recommendation. ADAMTS13 inhibitor assays are used to detect and quantify anti-ADAMTS13 autoantibodies that functionally inhibit ADAMTS13 activity in vitro. Inhibitor titers were determined using a mixing assay. Briefly, heat-inactivated patient plasma (56 °C for 60 min) was mixed with normal donor plasma at different ratios (1:1, 2:1, 3:1, and 4:1) and incubated for 2 h. Residual ADAMTS13 activity was subsequently measured, and inhibitor titers were calculated and expressed in BU [31,32].

In our center, 61 unrelated patients from different regions of Russia (Moscow, Saint Petersburg, Moscow Oblast, Bryansk Oblast, Saratov Oblast, Tomsk Oblast, Sverdlovsk Oblast, Krasnoyarsk Krai, Krasnodar Krai, Stavropol Krai and Udmurtia), with TTP of unknown origin were observed.

For the purposes of this study, we defined the following patient inclusion criteria: (1) absence of detectable ADAMTS13 inhibitors with ADAMTS13 activity < 40%; and (2) presence of ADAMTS13 inhibitors with ADAMTS13 activity < 10%. These criteria were selected in light of prior reports. Specifically, Subhan et al. [30] described a case of cTTP with relatively high ADAMTS13 activity (24–44%), while Camilleri et al. [15] demonstrated that in some cases, a combination of ADAMTS13 inhibitors and pathogenic genetic variants is possible.

**Figure 3:**
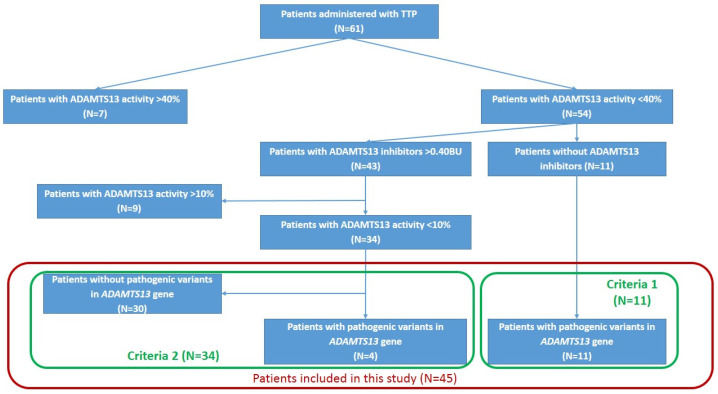
Patient selection scheme for the study. Fifty-four patients had reduced ADAMTS13 activity below 40% (range: 0–32%; normal ADAMTS13:C > 40%), while 7 patients had activity levels above 40%. Among those with decreased ADAMTS13 activity, more than half (43 out of 54, or 79.6%) had detectable ADAMTS13 inhibitors (0.6–95 BU), whereas the remaining 11 patients showed no evidence of inhibitors. Based on these findings, we defined our study cohort as follows: 11 patients with ADAMTS13 activity below 40% and no detectable inhibitors, and 34 patients with inhibitors and ADAMTS13 activity below 10%. In total, the final study group included 45 patients.

Based on these criteria, 45 patients were included in the final analysis: 11 patients with ADAMTS13 activity < 40% and no detectable inhibitors, and 34 patients with inhibitors and ADAMTS13 activity < 10% (Figure 3).

We performed molecular–genetic analysis for all patients in our sample. Genomic DNA was isolated from EDTA-preserved whole blood samples using phenol–chloroform extraction and ethanol precipitation. For full-scale mutational analysis, all functionally important regions of the *ADAMTS13* gene (promoter, all 29 exons and exon/intron junctions) were amplified as 23 fragments from 180 to 3780 bp in length (Table S1).

Oligonucleotide primers were designed based on the known sequence of the *ADAMTS13* gene (GenBank # NG_011934.2) and synthesized at Syntol JSC (Moscow, Russia). Amplification was carried out in a GoTaq^®^ Flexi DNA Polymerase PCR system (Promega Corporation, Madison, WI, USA) with 0.01–0.02 μg of genomic DNA and 10 pmol of each primer under average conditions (94 °C—1 min, 60–62 °C—1 min, 72 °C—3 min, 35 cycles). The longest fragment AD1011D/AD12R was amplified with long distance polymerase chain reaction (LD-PCR) using Promega GoTaq^®^ Long PCR Master Mix (Promega Corporation, Madison, WI, USA) with 0.01–0.02 μg of genomic DNA and 10 pmol of each primer under following conditions (warming up to 94 °C—2 min, then 94 °C—1 min, 60 °C—1 min, 72 °C—8 min, 35 cycles).

Direct Sanger sequencing of purified PCR products (using Wizard^®^ PCR Preps DNA Purification System (Promega Corporation, Madison, WI, USA)) was performed using BigDye Terminator Cycle Sequencing Kit ver.3.1 (Applied Biosystems, Foster City, CA, USA) and analyzed using an automatic genetic analyzer ABI PRISM 3100Avant (Applied Biosystems, Foster City, CA, USA) at CCU “Genome” (Institute of Molecular Biology, Russian Academy of Sciences, Moscow, Russia). Found nucleotide variants were named according to HGVS recommendations [33] using the *ADAMTS13* transcript NM_139025.5 as a reference. The detected variants were cross-referenced with available databases (HGMD and ClinVar [14,34]), while novel variants were assessed for pathogenicity following the ACMG/AMP Curation Guidelines and their updates [35].

Pairwise linkage disequilibrium (LD) between c.3178 C>T (p.Arg1060Trp) and c.3097 G>A (p.Ala1033Thr) was calculated from the genotype data and measured as D’. Statistical analyses were performed using R Statistical Software (v.4.3.0) [36]. LD was calculated using the package “genetics” [37].

## Figures and Tables

**Table 1:** Characteristics of patients included in the study and *ADAMTS13* gene variants.

#	Lab Code	Age	Sex	ADAMTS13 Activity (%) (Normal: >40%)	ADAMTS13 Inhibitor (BU *) (Normal: <0.4 BU)	Pathogenic Variant	Pathogenic Variant’s Zygosity	Polymorphisms
1	AD2	36	M	3.0%	0.6 BU	c.704 A>G (p.Asp235Gly) c.3975dup (p.Glu1326ArgfsTer6)	Heterozygous compound	c.1342 C>G (p.Gln448Glu) †
2	AD4	21	F	7.0%	0 BU	c.3178 C>T (p.Arg1060Trp) c.3975dup (p.Glu1326ArgfsTer6)	Heterozygous compound	c.3097 G>A (p.Ala1033Thr)
3	AD6-1	11	M	0%	0 BU	c.3198_3199del (p.Cys1067SerfsTer30) c.3975dup (p.Glu1326ArgfsTer6)	Heterozygous compound	c.1342 C>G (p.Gln448Glu)
4	AD8	31	F	3.0%	0.6 BU	c.1159 G>C (p.Trp387Ser) c.3178 C>T (p.Arg1060Trp)	Heterozygous compound	c.19 C>T (p.Arg7Trp) c.1342 C>G (p.Gln448Glu) c.3097 G>A (p.Ala1033Thr)
5	AD22	23	F	12.0%	0 BU	c.3178 C>T (p.Arg1060Trp)	Homozygous	c.3097 G>A (p.Ala1033Thr) †
6	AD24	34	M	10.0%	0 BU	c.1261 C>T (p.Arg421Cys) c.3178 C>T (p.Arg1060Trp)	Heterozygous compound	c.19 C>T (p.Arg7Trp) c.347 G>C (p.Arg116Pro) c.1342 C>G (p.Gln448Glu) c.3097 G>A (p.Ala1033Thr)
7	AD26	30	F	3.0%	0 BU	c.3975dup (p.Glu1326ArgfsTer6)	Homozygous	-
8	AD31	34	F	6.2%	0 BU	c.1492C>T (p.Arg498Cys) c.2074 C>T (p.Arg692Cys)	Heterozygous compound	-
9	AD34	38	F	0.0%	0 BU	c.1461 C>A (p.Ser487Ter) c.3975dup (p.Glu1326ArgfsTer6)	Heterozygous compound	c.1342 C>G (p.Gln448Glu)
10	AD38	25	F	0%	0 BU	c.3198_3199del (p.Cys1067SerfsTer30) c.1100 C>G (p.Ser367Cys)	Heterozygous compound	c.1342 C>G (p.Gln448Glu) †
11	AD41	18	M	12%	0 BU	c.414+2T>A c.3975dup (p.Glu1326ArgfsTer6)	Heterozygous compound	c.19 C>T (p.Arg7Trp)
12	AD43	21	F	4.6%	0 BU	c.746T>A (p.Met249Lys) c.3178 C>T (p.Arg1060Trp)	Heterozygous compound	c.19 C>T (p.Arg7Trp) c.1342 C>G (p.Gln448Glu)
13	AD48	34	F	5%	0.7 BU	c.987+5G>A c.3178 C>T (p.Arg1060Trp)	Heterozygous compound	c.19 C>T (p.Arg7Trp) c.3097 G>A (p.Ala1033Thr)
14	AD59	20	F	3%	0 BU	c.1627G>T (p.Asp543Tyr) c.3178 C>T (p.Arg1060Trp)	Heterozygous compound	c.3097 G>A (p.Ala1033Thr)
15	AD62	33	F	0%	0.6 BU	c.3198_3199del (p.Cys1067SerfsTer30) c.3975dup (p.Glu1326ArgfsTer6)	Heterozygous compound	c.1342 C>G (p.Gln448Glu) †

* Bethesda units; † Polymorphisms in homozygosity.

**Table 2:** *ADAMTS13* gene variants revealed in the study.

Location	DNA	Protein	Domain	dbSNP	ACMG Criteria	Reference
Causative variants						
ex4	c.414+2T>A	-	Metalloprotease	-	Likely Pathogenic (PM2, PVS1)	new
ex7	c.704 A>G	p.Asp235Gly	Metalloprotease	-	Likely Pathogenic (PM2, PM5, PP3, PM1, PP4)	new
ex7	c.746T>A	p.Met249Lys	Metalloprotease	-	Likely Pathogenic (PM2, PP3, PM1, PP4)	new
Ex8	c.987+5G>A	-	Disintegrin	-	VUS (PM2, PP3)	new
ex10	c.1100 C>G	p.Ser367Cys	Disintegrin	rs782270618	Likely Pathogenic (PM2, PP3, PM1, PP4)	new
ex10	c.1159 G>C	p.Trp387Ser	TSP1-1	-	Pathogenic (PM2, PM1, PP4)	[16]
ex11	c.1261 C>T	p.Arg421Cys	TSP1-1	rs145825553	Likely Pathogenic (PM2, PS3, PP4)	[13]
ex12	c.1461 C>A	p.Ser487Ter	Cysteine-rich	-	Likely Pathogenic (PM2, PVS1)	new
ex13	c.1492 C>T	p.Arg498Cys	Cysteine-rich	rs201457594	Likely Pathogenic (PM2, PP5)	[17]
ex14	c.1627 G>T	p.Asp543Tyr	Cysteine-rich	-	VUS (PP4, PM2, PP3)	new
ex17	c.2074 C>T	p.Arg692Cys	TSP1-2	rs121908475	Likely Pathogenic (PM2, PP5)	[1]
ex24	c.3178 C>T	p.Arg1060Trp	TSP1-7	rs142572218	Pathogenic (PM3, PP1, PM2, PS3)	[18]
ex24	c.3198_3199del	p.Cys1067SerfsTer30	TSP1-7	rs782288601	Pathogenic (PM3, PM2, PVS1)	[1]
ex29	c.3975dup	p.Glu1326ArgfsTer6	CUB-2	rs387906343	Pathogenic (PM3, PS3, PM2, PVS1)	[19]
Single nucleotide polymorphisms (SNPs)						
ex1	c.19 C>T	p.Arg7Trp	Signal peptide	rs34024143	Benign (BA1, BS2, BP4, BP6)	[1]
ex4	c.347 G>C	p.Arg116Pro	Metalloprotease	-	VUS (PM2, PP3)	new
ex12	c.1342 C>G	p.Gln448Glu	Cysteine-rich	rs2301612	Benign (BA1, BS2, BP4, BP6)	[1]
ex16	c.1852 C>G	p.Pro618Ala	Spacer	rs28647808	Benign (BA1, BS2, BP6)	[1]
ex21	c.2699 C>T	p.Ala900Val	TSP1-5	rs685523	Benign (BA1, BS2, BP4, BP6)	[1]
ex24	c.3097 G>A	p.Ala1033Thr	TSP1-7	rs28503257	Benign (BA1, BS2, BP6)	[1]

## Data Availability

The data that support the findings of this study are available on request from the corresponding author.

## Supplementary Materials

The following supporting information can be downloaded at: https://www.mdpi.com/article/10.3390/ijms27104643/s1.

## Abbreviations

The following abbreviations are used in this manuscript:

HGVS	Human Genome Variation Society (nomenclature system)
HGMD	Human Gene Mutation Database
ACMG	American College of Medical Genetics and Genomics
gnomAD	Genome Aggregation Database
dbSNP	The Single Nucleotide Polymorphism Database
